# An Arg124His mutation in *TGFBI* associated to Avellino corneal dystrophy in a Chinese pedigree

**Published:** 2011-12-13

**Authors:** Zhensheng Gu, Peiquan Zhao, Guang He, Chunling Wan, Gang Ma, Ling Yu, Juan Zhang, Guoyin Feng, Lin He, Linghan Gao

**Affiliations:** 1 Department of Ophthalmology, Xinhua Hospital, Medical College of Shanghai Jiao Tong University, Shanghai, China; 2 Bio-X Institutes, Key Laboratory for the Genetics of Developmental and Neuropsychiatric Disorders (Ministry of Education), Shanghai Jiao Tong University, Shanghai, China; 3 Department of Ophthalmology, Affiliated Hospital, Luzhou medical college, Luzhou, Sichuan, China; 4 Institute for Nutritional Sciences, Shanghai Institute of Biological Sciences, Chinese Academy of Sciences, Shanghai, China; 5 Institutes of Biomedical Sciences, Fudan University, Shanghai, China

## Abstract

**Purpose:**

To identify the gene mutation underlying Avellino corneal dystrophy in a four-generation Chinese pedigree.

**Methods:**

Patients from the affected family underwent detailed clinical examination involving slit-lamp photography and confocal microscopy. Genomic DNA extracted from peripheral leukocytes was amplified using touch-down PCR for gene scanning. Two-point linkage analysis and haplotyping were performed to map the relevant chromosome region. The candidate gene in this region was sequenced to screen out the disease-causing mutation.

**Results:**

Patients in the pedigree were diagnosed with Avellino corneal dystrophy. Using linkage analysis, the responsible gene was mapped to chromosome 5q31.2 with a maximum LOD (log odds) score (*Z*_max_) of 3.23 at D5S479 (θ_max_=0.0). Haplotypes constructed from 11 microstallite markers identified the disease-linked chromosome region as being below D5S808. Sequencing of *TGFBI* (transforming growth factor-beta induced gene), a known gene in this region, revealed a heterozygous transition (c.418 G>A) in exon 4 resulting in Arg124His (R124H) being co-segregated with the disease in affected family members but not in the unaffected members or the 50 unrelated controls.

**Conclusions:**

Our study demonstrated that a G>A transition in Arg124His of *TGFBI* was responsible for Avellino corneal dystrophy in a Chinese pedigree. This result further supports the importance of TGFBIp in maintaining transparency of the cornea.

## Introduction

Corneal dystrophy is a heterogeneous inherited disease with bilateral, symmetric, non-inflammatory, progressive cornea opacities which lead to varying degrees of visual impairment. Clinical examination using slit-lamp photography, confocal microscopy and histological staining, enable corneal dystrophies to be categorized as epithelial, sub-epithelial, Bowman’s layer, stromal, and endothelial according to the affected layer in the cornea. More than twenty types of corneal dystrophy have so far been categorized based on disease features including physical appearance, age of onset and histological tests [1]. Historically, clinical classification of corneal dystrophy has been based entirely on ophthalmological and histopathologic examination but these have proved to be limited in scope and effectiveness [2].

More recently the development of molecular genetics has enabled isolated corneal dystrophy to be linked to autosomal dominant, autosomal recessive or X-linked recessive Mandelian inheritance traits even where penetrance is variable. Most of the genes responsible for corneal dystrophies can be identified by pedigree analysis including linkage analysis, haplotyping and direct sequencing based on the particular corneal dystrophy affecting the group. Individual mutations related to a particular disorder have often been reported in families of varying origins. Genetic studies have identified eleven chromosomes related to corneal dystrophy: 1, 2, 5, 9, 10, 12, 13, 16, 17, 20, and X. Several genes in regions responsible for corneal dystrophy have also been identified, including the transforming growth factor-beta-induced gene (*TGFBI*), the carbohydrate sulfotransferase 6 gene (*CHST6*), the gelsolin gene (*GSN*), the keratin 3 gene (*KRT3*), the keratin 12 gene (*KRT12*) and the surface marker 1 gene (*M1S1*) [1,3].

Increasing genetic knowledge, as well as revealing the molecular basis of corneal dystrophy has also raised questions relating to phenotypic and genetic heterogeneity aspects of the disease. Studies have revealed that different phenotypes of corneal dystrophy share a common gene or mutation in the same site, or even the same mutation. The most powerful evidence has come from *TGFBI*-related corneal dystrophies. Different types of corneal dystrophy have been categorized including granular corneal dystrophy (GCD) and lattice corneal dystrophy (LCD) [4]. As a special critical mutation, Arg124His of TGFBI corresponds to several subtypes of GCD and LCD in families of different ethnic origin while different mutations in the same genes or even the same mutation can result in a single phenotype. For example, two distinct mutations Arg124Ser and Arg555Trp have been found to cause the common subtype of GCD, namely granular corneal dystrophy type I (GCD1) [4]. The complexity of phenotypic and genetic heterogeneity therefore complicates the identification of the causes of the disease.

The challenge to ophthalmologists and researchers is to correctly diagnose and classify corneal dystrophies, as well as to understand their phenotype-genotype aspects. In 2008, a useful nomenclature for corneal dystrophies was established by the International Committee on the Classification of Corneal dystrophies (IC3D) based on traditional clinical examination and advances in molecular genetics. Under the IC3D classification system, each dystrophy has a detailed description including an OMIM number, eponyms, genetic loci, relevant genes, onset, signs, symptoms, histopathology, etc., combining clinical features and genetic characteristics including molecular mechanism and protein functions [5-7].

In this study, we recruited a Chinese four-generation pedigree affected by corneal dystrophy and identified the gene mutation responsible for the disease. We hope our study will provide an insight into the mechanisms of the disease.

## Methods

### Clinical examinations

This research was approved by the ethics committee of Shanghai Xinhua Hospital in Shanghai, China. Twenty-three members in a four-generation Chinese pedigree in Han population with corneal dystrophy were recruited for the study. Informed consent was signed in accordance with the Helsinki Declaration by all participants. Full family medical history was recorded. Participating members underwent careful ophthalmic examination including visual acuity tests, slit-lamp examination and confocal microscopy (Heidelberg retinal tomography; HRT-II, Heidelberg, Germany). Fifty unrelated subjects without corneal dystrophy were recruited from the Ophthalmology Clinic of Xinhua Hospital as normal controls for the study.

### Genescan and genotyping

Genomic DNA was extracted from peripheral leukocytes using the QIAamp Blood Mini DNA kit (Qiagen, Santa Clara, CA). Genescanning was performed using microsatellite markers based on the NCBI span of known genes related to granular corneal dystrophy. Genotyping was performed using capillary electrophorese of the touchdown PCR mixture.

A three-temperature touchdown PCR program was used for DNA amplifying: 96 °C for 12 min; 14 cycles at 95 °C for 30 s, 64 °C for 30 s (with temperature decreasing from 64 °C to 57 °C by −0.5 per cycle), 72 °C for 30 s; additional 30 cycles at 95 °C for 30 s, 57 °C for 30 s, 72 °C for 30 s; and a final extension at 72 °C for 10 min, using the Gene Amp PCR System 9700 (PE Applied Biosystems, Foster City, CA).

Standard PCR reactions wes preformed in a 5 μl volume containing 20 ng template DNA, 0.1 μM of each primer, 300 μM dNTP, 1 µl of 10× PCR buffer, 7 mM MgCl_2_, and 0.3 U Hotstart Taq.

The PCR products were mixed according to size (Genescan-400HD ROX; Perkin Elmer, Foster City, CA), denatured at 95 °C for 1 min and electrophoresed on a 96-capillary automated DNA sequencer (MegaBACE 1000; Amersham, Freiburg, Germany). Genotyping data were analyzed on Genetic Profiler software (version 1.5, Amersham).

### Linkage analysis and haplotyping

Genotyping data were calculated using the MLINK subprogram from the LINKAGE package (version 5.1) for two-point linkage analysis. Microsatellite markers, allele frequencies and recombination distances between the marker loci were based on the Marshfield Database and the UCSC Database. Autosomal dominant inheritance, disease-gene frequency of 0.0001 and full penetrance were assumed. Cyrillic (version 2.1) software was used for haplotype construction.

### Mutation sequencing

*TGFBI*, the relevant gene for corneal dystrophy in the defined region was sequenced to screen the mutation in coding exons, splice regions and UTRs. Sequencing reactions were performed using the BigDye Terminator Cycle Sequencing Kit v3.1 (Applied Biosystems, Foster City, CA) on an ABI PRISMTM 3730xl analyzer. Sequencing data were analyzed using Sequence Scanner v1.0 software referencing the NCBI GeneBank (NM_000358 for *TGFBI*). Gene mutation was confirmed in all family subjects and unrelated controls.

## Results

### Clinical features

**Figure 1:**
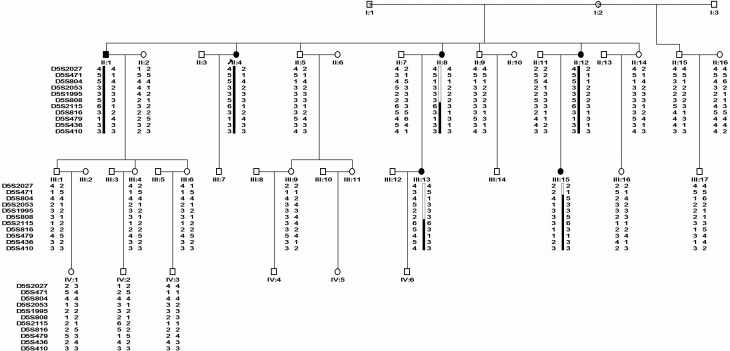
Pedigree and haplotyping of the family with Avellino corneal dystrophy. Squares and circles indicate males and females, respectively; affected individuals are shaded black; An arrow denotes the proband. Black and white bars depict the disease and non-disease associated haplotypes respectively. Haplotyping with STR markers harbored the causative gene located below D5S808.

The twenty-three members (six affected and seventeen unaffected individuals) participating in the study were recruited from a four-generation Chinese pedigree that consist of forty-two individuals including the six patients (Figure 1). Affected individuals in the family were having low myopia decreased vision of both eyes but had no other ocular abnormalities. Their best corrected visual acuity (BCVA) was 20/60–20/30. Even intraocular pressure was normal with 18.4±2.3 mmHg on the right eye and 17.6±1.8 mmHg on the left eye, which indicated that corneal dystrophy in the family didn’t result in corneal edema. Proband (II:4) and her siblings (II:1, II:8, and II:12) had only slow vision decrease but had no other ocular problems when growing up and had been diagnosed with corneal dystrophy after the age of 48. Two of those affected in the next generation, the nieces (III:13 and III:15) of the proband, had had obvious visual impairment as well as symptoms of gradual photophobia, lacrimation, dry eye, and foreign-body sensation by the age of 30 and 25, respectively. Further ophthalmic examination showed that they had recurrent corneal epithelial erosion which was susceptible to relief with anti-inflammatory treatment, cornea re-epithelialization, and the use of artificial tears.

**Figure 2:**
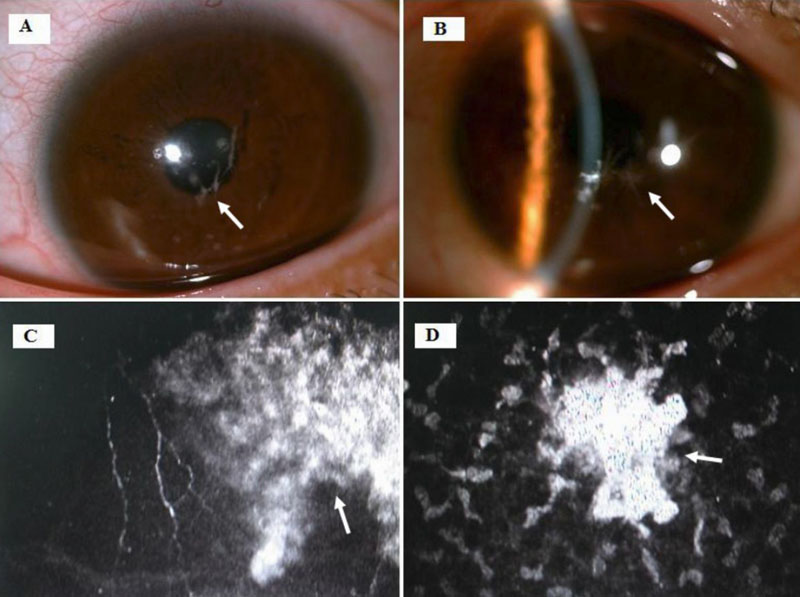
Slit-lamp photographs showing granular deposits distributed in the left eye (**A**, individual III:13; **B**, individual III:15). The arrow indicates liner opacities in the superficial stroma. Confocal images showing numerous hyper-reflective dots with sharp shapes scattered between stromal cells and nerve fibers in the superficial corneal stroma in the left (**C**) and right eye (**D**) both of individual III:13.

Slit lamp examination showed a gray white deposit located beneath the central cornea, involving the Bowman layer and superficial stroma. Opacities initially appeared granular, snow-flake, star or branch shaped but gradually coalesced to form a circle or irregular block (Figure 2). Visual impairment of Individual II:1, the elder brother of the proband was less than that of the proband (II:4) and her sisters (II:8 and II:12). Corneal dystrophies in individuals III:13 and III:15 were worse than in the older generation and exhibited punctate corneal epithelial shedding. Numerous hyper-reflective dots with sharp shapes were scattered in the superficial corneal stroma, mostly between stromal cells and nerve fibers. Their diameter covered dozens of stromal cells (Figure 2). Affected individuals in the family were diagnosed as having granular corneal dystrophy type II (GCD 2) – Avellino corneal dystrophy.

### Linkage and haplotype analysis

Indicative results were first observed on chromosome 5 in the screening of granular corneal dystrophy related loci. Several markers were then selected for further study, and positive LOD (log odds) scores were obtained, including the maximum LOD score (*Z*_max_) of 3.23 at D5S479 (*θ*_max_=0.00; Table 1). Haplotyping revealed three informative recombination events occurring in the family. Crossover occurred in II:15 suggesting that the proximal border was between D5S471 and D5S804. The other two recombinations occurred in individuals II:8 and III:13 where the proximal border was narrower at between D5S808 and D5S2115. We therefore concluded that the chromosome region responsible for corneal dystrophy in this family is below D5S808.

**Table 1:** Two point LOD scores for autosomal dominant Avellino corneal dystrophy on chromosome 5p.

** **	** **	**LOD score at θ=**					** **	** **
**Marker**	**Mb**	**0.0**	**0.1**	**0.2**	**0.3**	**0.4**	***Z*_max_**	***θ*_max_**
D5S2027	111,045,419–111,245,714	−4.44	−0.83	−0.35	−0.11	−0.01	−0.01	0.4
D5S471	118,948,944–119,149,278	−3.62	−0.41	−0.22	−0.14	−0.07	−0.07	0.4
D5S804	124,984,914–125,185,374	−2.95	1.18	0.99	0.61	0.19	1.18	0.1
D5S2053	133,016,815–133,217,150	1.46	1.24	0.99	0.71	0.38	1.46	0.0
D5S1995	133,219,912–133,420,267	0.27	0.23	0.18	0.13	0.07	0.27	0.0
D5S808	133,533,511–133,733,691	−2.90	1.34	1.14	0.76	0.30	1.34	0.1
D5S2115	134,619,248–134,819,548	2.93	2.39	1.79	1.11	0.38	2.93	0.0
D5S816	135,201,390–135,401,753	1.44	1.21	0.97	0.69	0.37	1.44	0.0
D5S479	136,205,608–136,405,941	3.23	2.70	2.09	1.41	0.65	3.23	0.0
D5S436	145,103,918–145,304,281	0.57	0.49	0.39	0.27	0.15	0.57	0.0
D5S410	152,674,975–152,875,361	1.59	1.28	0.94	0.56	0.19	1.59	0.0

### Mutation analysis

**Figure 3:**
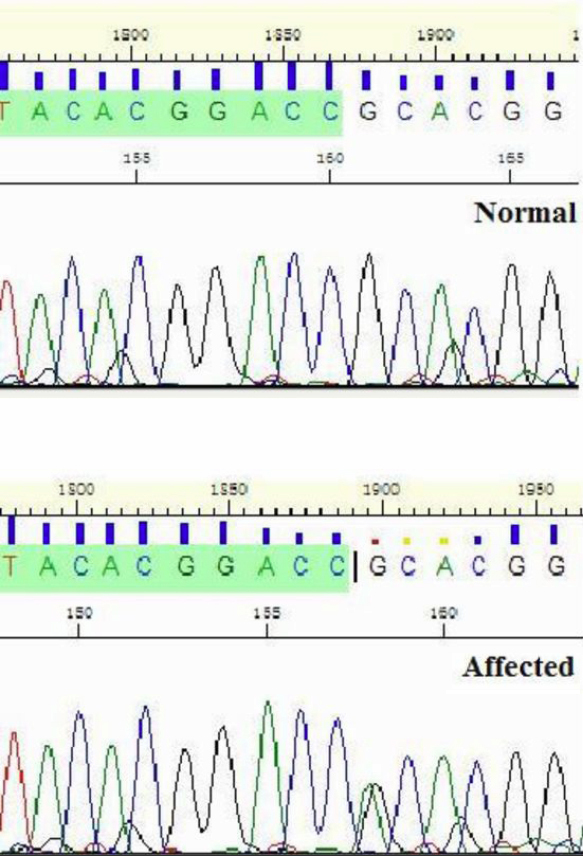
Sequence analysis of the Chinese pedigree with Avellino corneal dystrophy. Position c.418 G>A transition (indicated by the arrow) resulting in Arg124His (R124H) co-segrated with all patients in the family, but was not found in the unaffected family members nor in the 50 unrelated control subjects.

The gene *TGFBI* (bp: 135,364,584–135,399,507) which is known to be related to granular corneal dystrophy, is exactly below D5S808 (bp: 133,533,511–133,733,691) and was sequenced first. Direct sequencing of the UTRs, exons, and exon/intron boundaries were performed in affected and unaffected family members and 50 normal controls (sequencing primers are presented in Table 2). A heterozygous c.418 G>A mutation in exon 4 of *TGFBI* was identified in all affected individuals but not in unaffected members or controls. This mutation led to a substitution at codon 124 (Arg124His, R124H) which is responsible for Avellino corneal dystrophy in this family (Figure 3).

**Table 2:** Primers designed for sequencing of the *TGFBI* gene.

**Primer for exon**	**Forward**	**Reverse**	**Length (bp)**
1	caggaggcctaagggaccta	ctccatgctgcaaggttttt	607
2	tcaattgcccatgtcaaaga	gccctgaaaaatgtctccaa	607
3	ccagttggttggctgtaggt	gaggagcagctcaggaaatg	514
4	ccccagaggccatccctcct	ccgggcagacggaggtcatc	358
5	ggcatgatgaatgggagtct	gagaagcaggcacaaagagg	579
6	tctccttgggccctctatt	tcaggggaacctgctctatg	416
7	aggaagaggaaaggcaggtt	agcaacaggacaggatgacc	532
8	agaaggcgaggaggatctg	gtcacaacccacacatttgc	527
9	tgactgttcccctgatgaca	ttttggttgagctgagtgga	434
10	ttggcagcttcacttggttt	ttccttccttgtcagcaacc	409
11	tcccagccttaataacccatc	cttttccccatcccaagtct	433
12	tccagtggcctggactctac	gatgtgccaactgtttgctg	337
13	tgctttgtgtcctctgacca	catcctgggggtgagatatg	402
14	ggcgacaagattgaaactcc	cccaattcactctgcaatca	405
15	tgtgcattcacctttcttgg	agtgggagtggggagaagtt	406
16	gtccacctgaaggcacactt	ccaagtcaccctgctgttct	393
17	cacctgctatgtgcaggaga	ggctggattgcttgattcat	532

## Discussion

As the most common type of corneal dystrophy, granular corneal dystrophy involves the anterior corneal stroma and can be divided into three subtypes. Granular corneal dystrophy type I (GCD1, OMIM 121900), also know as classic granular corneal dystrophy, presents ‘bread-crumb-like’ white well defined granular opacities within a crystal clear cornea and onset is usually in the first decade of life. The onset of Granular corneal dystrophy type II (GCD2, OMIM 607541), also known as Avellino corneal dystrophy (ACD), often occurs in the second decade and demonstrates fewer opacities than GCD1 with granular, branching deposits in a clear superficial mid stroma and lattice lines sometimes in deeper corneas. Visual acuity in GCD2 is less impaired than in GCD1 because of the slower progression of the disease. Granular corneal dystrophy type III (GCD3, OMIM 608470), also called Reis-Bücklers corneal dystrophy (RBCD), has been found to exhibit opacities in irregularly ring-shaped spots and lines in the superficial cornea. In the present genetic study, we recruited a four-generation Chinese pedigree having corneal dystrophy. Slit-lamp photography and confocal microscopy examination showed affected members in the family to have granular corneal dystrophy type II, namely Avellino corneal dystrophy. The disease gene was mapped to 5q31, the exact location of *TGFBI*. Direct sequencing then revealed that the c.418 G>A mutation of *TGFBI* resulting in Arg124His was responsible for Avellino corneal dystrophy in the pedigree. Our result further supports the importance of TGFBIp in maintaining corneal transparency.

*TGFBI*, the transforming growth factor-beta induced gene (also known as *BIGH3*, OMIM 601692), was initially observed to be inducibly expressed in the human lung adenocarcinoma cell line A549 and in several other cell lines in 1992 [8]. Two years later, TGFBI protein (TGFBIp) was found expressed in the cornea [9] and its chromosome locus found to be involved in corneal dystrophy [10]. In 1997, Munier et al. [11] first reported that *TGFBI* mutations were related to corneal dystrophy. *TGFBI* comprises 17 exons and encodes a 68 kDa extracellular matrix protein (TGFBIp, kerato-epithelin, KE) which is expressed in the cornea, skin, bone, tendon, kidney and other connective tissues. TGFBIp comprises 683 amino acids and contains an N-teminal signal peptide (Met1-Ala23), four internal homologous repeat domains and a highly conserved COOH-terminal sequence that is the integrin-binding motif RGD (Arg642-Gly643-Asp644). The NH_2_-terminal sequence contains a cysteine-rich EMI domain (Gly45-Ala99) which was initially recognized in EMILINs [12]. Residue Ala100-Pro635 is composed of four fasciclin (FAS) domains consisting of 140 amino acids each fasc1–4. FAS-containing proteins play an important role in extracellular or membrane binding in cell adhesion. Two conserved sequences, Asn-Lys-Asp-Ile-Leu within fasc 2 and Glu-Pro-Asp-Ile-Met within fasc 4 have been identified as mediating cell adhesion. Studies have shown that not all four domains (fasc1–4) are equally important in mediating the normal activity of TGFBIp. Fasc1 and fasc 4 play more important roles than other two fasc domains, though their specific interaction with other unknown proteins is still unclear [13,14]. COOH-terminally processed and matured corneal TGFBIp isoform. RGD is known to act as a ligand recognition sequence for integrins in modulating cell adhesion within the extracellular matrix (ECM). Ser658-His683 in the C-terminal is critical for function malignance of RGD because the lack of these residues is liable to expose the RGD sequence to interactions [15,16]. TGFBIp widely expresses in the extracellular matrix of many organs and interacts with other matrix proteins, such as fibronectin, different integrins, and collagens [17-19].

Numerous studies have indicated that *TGFBI* is the important disease gene underlying corneal dystrophy. Mutations of *TGFBI* mainly occur in exon 4, 11, 12, and 14. Up to now, more than 50 mutations in *TGFBI* have been proved to be associated with corneal dystrophy, including epithelial basement membrane corneal dystrophy (EBMD, OMIM 121820), Bowman’s layer dystrophies: Thiel-Behnke corneal dystrophy (TBCD, OMIM 602082), Reis-Bücklers corneal dystrophy, and the stromal dystrophies: lattice corneal dystrophy type I (LCD I, OMIM 122200), GCD1, and GCD2.

Mutations occur in two arginine codons, Arg124 and Arg555, which account for more than half of TGFBI mutations in corneal dystrophy, indicating that these two sites are mutant hotspots of *TGFBI*. Studies suggest that these two mutations are likely to affect protein–protein interactions in TGFBI-mediated cell-adhesion. Arg124, along with two valines at positions 112–113, as well as disulfide and hydrogen bonding, is the most important factor for amyloid formation of TGFBIp [20]. Four different mutations in this position have been linked with corneal dystrophies in families worldwide [21]: transition c.417 C>T leading to Arg124Cys (R124C) has been identified in GCD2 [22] and LCD1 [23,24]; transversion c.417 C>A resulting in Arg124Ser (R124S) has been found to be related to a phenotypic variant of GCD1 [25]; transition c.418 G>A is known to cause Arg124His (R124H) in GCD2, or a mixed type of amyloid and granular deposits [26]; while transversion c.418 G>T resulting in Arg124Leu (R124L) has been reported in GCD3, a phenotypic variant of GCD1 characterized by superﬁcial granular deposits [27,28]. Nucleotides CGG (c.1710–1712) in exon 12 encode amino acid Arg555 in the fasc-4 domain. Transition c.1710 C>T resulting in Arg555Trp (R555W) is associated with GCD1 [26] or the phenotype similar to GCD1 and transition c.1712 G>A resulting in Arg555Gln (R555Q) is responsible for Thiel-Behnke corneal dystrophy [29,30].

TGFBIp is known to bind collagen microfibrils with covalent and mediates the adhesion of various cell types. In the cornea, TGFBIp is preferentially expressed on the extracellular surface of corneal epithelial cells [31]. EMI, fasc1–4 and RGD are critical for TGFBIp in cell-collagen interactions with a role in the regulation of cell-adhesion in maintenance of tissue architecture. Fasc1 is important in mediating the normal activity of TGFBIp for FAS-containing proteins play an important role in extracellular or membrane binding in cell adhesion. We consider that change of Arg124 in fasc1 may effect on the structure formation or functional performing of TGFBIp,which indicated Arg124 is a conserved amino acid for TGFBIp. It is interesting that though TGFBIp expresses in many organ, such as skin, bone, kidney and so on, manifestations of its mutation have been found only in the cornea [32]. How mutations of TGFBI cause corneal dystrophy is still unknown. Researches including gene functional study are needed for further confirmation. Factors such as age, environment, and modifier genes may influence the expressivity and penetrance of corneal dystrophy. Despite of this, genetic studies of corneal dystrophy are still not only useful for ophthalmologist in clinical diagnosis, but can also help future research into the molecular mechanism underlying the disease.
